# Non-Linear Signal Detection Improvement by Radiation Damping in Single-Pulse NMR Spectra

**DOI:** 10.1002/cphc.201100724

**Published:** 2012-01-20

**Authors:** Judith Schlagnitweit, Steven W Morgan, Martin Nausner, Norbert Müller, Hervé Desvaux

**Affiliations:** [a] Institute of Organic Chemistry, Johannes Kepler UniversityAltenbergerstraße 69, 4040 Linz (Austria) Fax: : (+43) 732 2468 8747; [b] CEA, IRAMIS, SIS2M, Laboratoire de Structure et Dynamique par Résonance MagnétiqueUMR CEA/CNRS 3299, CEA/Saclay, 91191 Gif-sur-Yvette (France), Fax: (+33) 16908 2199; c Ecole Normale Supérieure; CNRSUPMC 24 rue Lhomond, F-75005 Paris (France)

**Keywords:** analytical methods, cross-precession, magnetic properties, NMR spectroscopy, radiation damping

## Abstract

When NMR lines overlap and at least one of them is affected by radiation damping, the resonance line shapes of all lines are no longer Lorentzian. We report the appearance of narrow signal distortions, which resemble hole-burnt spectra. This new experimental phenomenon facilitates the detection of tiny signals hidden below the main resonance. Theoretical analysis based on modified Maxwell–Bloch equations shows that the presence of strong transverse magnetization creates a feedback through the coil, which influences the magnetization of all spins with overlapping resonance lines. In the time domain this leads to cross-precession terms between magnetization densities, which ultimately cause non-linear behavior. Numerical simulations corroborate this interpretation.

## 1. Introduction

Radiation damping is an effect known since the early days of NMR.^1^ This non-linear phenomenon results from the interaction of the magnetization with itself, mediated through the tightly coupled detection coil. Radiation damping is nowadays routinely encountered in high-resolution liquid-state NMR spectroscopy due to the increase of magnetization achieved by high static magnetic fields and/or hyperpolarized species and by the improvements of the electronic detection circuit, in particular by cold probes, which exhibit huge quality factors. Radiation damping is often seen as a spurious and undesired effect which broadens resonance lines for small flip-angle excitation pulses,^1^ distorts the line shape for larger flip angle pulses,^2^ and even prevents the safe and reliable inversion of longitudinal magnetization.^3^ As a consequence several NMR methods, such as WATERGATE,^4^ water flip back,^5^ presaturation,^6^ jump-and-return^7^ or electronic devices for controlling radiation damping^8^ have been developed either for taking benefit of the fast transverse decay rates of magnetization experiencing radiation damping or for suppressing its effects for intense signals from the acquired spectra. More recently the situation has changed. Instead of being considered as an unpleasant effect, that has to be avoided, its non-linear feature is used for experimental studies, either for exploring new physical phenomena such as chaotic behavior,^9^ potentially enhanced by electronic circuits,^10^ or multiple spontaneous maser emissions^11^ or for evaluating alternative detection schemes such as radiation-damping amplified signal detection^12^ or nuclear-spin noise.^13^–^15^ The present study belongs to this last class. Indeed we have experimentally observed that the line shapes of spin systems experiencing radiation damping and that have close resonance frequencies, are strongly affected and strongly differ from the usual bell-shaped curves observed for small excitation pulses. Indeed, they seem to be affected by a hole-burning phenomenon. We show that this effect results from additional cross-precession terms present in the spin dynamics, which couple the evolutions of different magnetization densities through the induction they create inside the coil. Thanks to these non-linear interactions, we show that tiny signals resonating within the line width of large ones become easily detectable, since they introduce strong narrow distortions into the line shape, which are amplified or look like “holes” in the main resonance line instead of the expected small positive peaks or shoulders.

## 2. Theory

### 2.1. Radiation Damping Field

**Figure 1:**
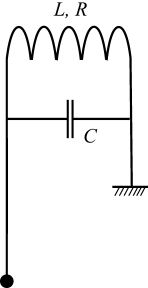
Classical probe with a coil of inductance *L*, resistance *R*, and with tuning capacitor *C*.

We consider the simple resonant electronic detection circuit depicted in Figure 1 and follow the derivation of Vlassenbroek et al. for computing the radiation damping field.^16^ We assume that the detection coil is perpendicular to the static magnetic field 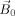, that we assume to be perfectly homogeneous. As a result, the transverse component of the precessing nuclear magnetization 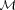
_*x*_(*t*) creates an electromagnetic force (emf) *V_s_*(*t*) in this detection coil, which can be calculated thanks to the reciprocity principle given by Equation (1):


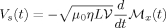
(1)

where *μ*_0_ is the the magnetic permeability of free space, *η* the filling factor, *L* the inductance of the coil, and 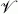 the sample volume. This emf creates a current *I*(*t*) in the idealized detection circuit [Eq. (2)]:


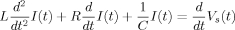
(2)

By introducing the characteristic parameters, 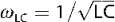 the resonant frequency of the detection circuit, and *Q*=*Lω*_LC_/*R*, the quality factor of the coil, the equation describing the flowing current as a function of the precessing transverse magnetization is obtained [Eq. (3)]:


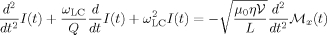
(3)

This differential equation corresponds to that of a damped harmonic oscillator driven by an external source. Transient phenomena are present for periods of the order of a few *Q*/*ω*_LC_, which typically represents a few tens of microseconds for liquid-state NMR probes with the highest *Q*. This is most of the time a short duration compared to the typical evolution time of magnetization (hundreds of milliseconds). We can consequently consider only the steady-state AC regime for the solution of Equâtion (4):


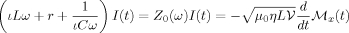
(4)

with 
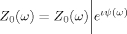
, the complex impedance [Eq (5)]:


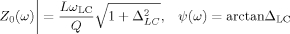
(5)

Δ_LC_ is a function of the offset between the Larmor frequency and the electronic circuit resonance frequency Eq. (6):


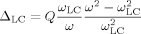
(6)

Finally the magnetic field 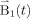 created by the precessing transverse magnetization is given by Equation (7):


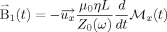
(7)

Using 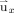, 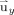, 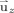 as the unit vectors of the respective coordinate axes, the equations describing the evolution of the nuclear magnetization density 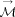 are obtained by standard theoretical approaches. Considering a single nuclear spin 1/2 system, which can be described by the Bloch equations, we have Equation (8):


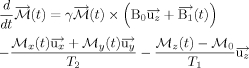
(8)

where *γ* is the magnetogyric ratio, and *T*_1_ and *T*_2_ are the longitudinal and transverse self-relaxation times. Equations (7) and (8) are the Maxwell–Bloch equations. Since 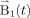 is dependent on the transverse magnetization, the evolution of the magnetization, as described by Equation (8) is no longer linear. These equations can be numerically integrated to explore the effects of distributions of Larmor resonance frequencies or the effects of a mis-tuned electronic circuit.^3^, ^9^, ^16^ Several studies have been reported in these cases and in particular an analytical solution for negligible longitudinal relaxation 1/*T*_1_=0 and perfect tuning (Δ_LC_=0) even exists whatever the initial flip-angle excitation pulse.^2^

### 2.2. Radiation Damping in the Presence of Two Resonance Lines

Herein we consider the existence of two different species of magnetization (ℳ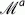 and ℳ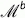) of different Larmor resonance frequencies (*γ*(1−*σ*^*a*^)*B*_0_ and *γ*(1−*σ*^*b*^)B_0_, where *σ*^*a*^ and *σ*^*b*^ are the shielding constants of ℳ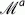 and ℳ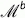, respectively). The principles used in the preceding derivation are still valid, and we can deduce that the magnetic field 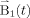 is now given by Equation (9):^1^


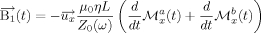
(9)

and the evolution of the magnetization, still considering independent spins 1/2, is obtained by inserting the new expression of 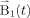 into Equation (8), to yield Equations (10) and (11):


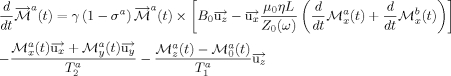
(10)


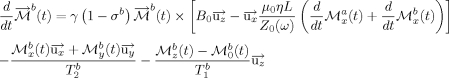
(11)

where straightforward extensions of the notations were used. These six differential equations [Eqs. (10) and (11)] are all coupled together through the radiation damping field [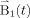, Eq. (9)], although we have considered independent spins. The evolution in the rotating frame is obtained by considering only one of the two rotating components of 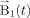. Finally in a frame rotating at *ω*_0_=*γ*B_0_, this coupling between the evolutions of the transverse magnetization densities of ℳ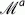 and ℳ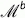 is readily illustrated by the evolution of one rotating component [Eq. (12)]:


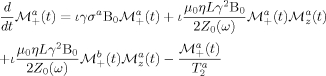
(12)

In this equation we recognize the Zeeman term, the classical radiation damping term, the cross-precession term due to feedback field and finally the transverse relaxation contribution. In summary, through the coupling to the detection coil cross-precession terms between the two magnetization densities appear.

*Z*_0_(*ω*) being a slowly varying function of *ω* around the Larmor resonance frequencies, since it depends on the half-width of the tuned electronic circuit at 3dB, [*ω*_LC_/(2*Q*)], can be considered as constant and the two contributions to the 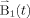 field, that is, the cross-precession terms, can be compared directly. Considering the ℳ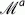 magnetization, the cross-precession term due to radiation damping resulting from the transverse magnetization of ℳ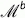 can be seen as an off-resonance rf excitation whose amplitude is proportional to ℳ
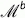
_*x*_ and whose frequency is equal, to a first approximation (see below), to the ℳ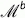 Larmor frequency, *γ*(1−*σ*^*b*^)B_0_. As usual for a cross-precession term between transverse components, it will be completely negligible, that is, non-secular, if the difference of resonance frequencies |*γ*(*σ*^*a*^−*σ*^*b*^)B_0_)| is much larger than the typical radiation damping characteristic rate 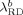 induced by ℳ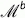 [Eq. (13)]:


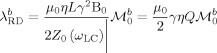
(13)

This corresponds to the maximal transverse decay rate [contribution from ℳ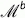 to the 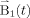 field], obtained for perfectly tuning Δ_LC_=0, and very small flip angles [Eq. (12)].^1^, ^2^ 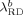 can be estimated from the full width at half maximum (FWHM in Hz) of the ℳ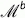 resonance after a small flip excitation pulse, since (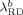
+1/*T*_2_^*b*^)=πFWHM. In summary, this cross-precession term has to be considered if the two resonance lines of ℳ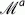 and ℳ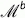 are overlapping or are close to this condition.

Although this semi-quantitative description provides the experimental conditions where cross-precession between two independent resonances due to radiation damping can be observed, the analytical prediction of the resonance line-shapes is prevented by the non-linear characters of Equations (10) and (11). Numerical simulations, however, can provide a detailed insight into the resonance line shapes.

### 2.3. Extension of the Theoretical Framework

Several comments lead us to extend the previous derivation to take into account more complex spin systems or other contributions neglected in a first approximation:

- The extension from two spins to *n* species is obtained by adding their contributions to the 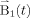 field [Eq. (9)] and by modifying the expression of Bloch equations [Eq. (10)] accordingly.
- As a first approach, for a simple 1D acquisition spectrum, a scalar-coupled *m*-multiplet can be described as a superposition of *m* fictitious spins 1/2 with appropriate intensities, the evolution of which follows the previous description.
- According to the tuning frequency of the coil *ω*_LC_, the angle *ψ*(*ω*) between the radiation damping field and the magnetization varies as well as the coupling between the magnetization and the coil [Eq. (5)].^14^, ^16^ This gives rise to a variation of the apparent radiation damping characteristic rate and to a “cavity pulling” effect or more rigorously to a frequency pushing.^14^ The latter leads to a variation of the central frequency of the line affected by radiation damping.^17^, ^18^ This frequency shift results from the imaginary component of *Z*_0_(*ω*), and is thus on the order of sin*ψ*(*ω*)*λ*_RD_, that is, up to a few hundreds rad s^−1^ for experiments performed with a cold probe.
- A strong magnetization creates an average dipolar field, which superimposes on the static magnetic field and is thus experienced by all spins.^19^ The amplitude of this average dipolar field is dependent on the *z* component of the magnetization, on the sample shape, and on a factor of 3/2 or 1 depending on the nature of the magnetization which creates the field (“like” or “unlike” spins^20^). These contributions should be added to the Bloch equations, and are typically time-dependent during the acquisition (a variation of the longitudinal magnetization potentially counterbalanced by the B_0_ feedback field controlled by the lock signal). The order of magnitude of these contributions (∼*μ*_0_*γ*ℳ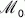) is less than 10 rad s^−1^ for a bulk solvent in a high magnetic field at room temperature. It can consequently easily be masked by cavity pulling (∼Δ_LC_*ηQ*/2×*μ*_0_*γ*ℳ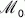). Herein, we neglect these average dipolar field contributions in the numerical simulations, in part because we used small flip-angle excitation pulses, so that ℳ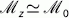 is nearly constant.
- Finally transverse and longitudinal dipolar cross-relaxations (and dynamic frequency shifts) between the different magnetization species can be included. These contributions are typically one tenth of the intermolecular dipolar self-relaxation contributions, that is, on the order of 0.1 Hz and consequently they have not been included in the numerical simulations, here. For systems of equivalent spins, in particular methyl groups, with long correlation times, cross-correlation effects^21^ may be larger and also need to be considered for further refinement.

## 3. Results and Discussion

**Figure 2:**
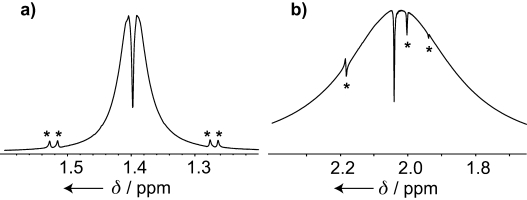
Example of non-linear response induced by radiation damping when two lines overlap. These spectra were acquired in conditions where cavity pulling vanishes [*ψ*(*ω*)=0] with small flip-angle excitation pulses. a) Case of the doublet of isopropyl acetate. This spectrum was acquired at 600 MHz with a cold probe. The solution contained 50 % vol/vol of isopropyl acetate. b) Case of a mixture of acetone and acetonitrile (40:5) with a concentration of 80 % in volume of acetone (lock solvent: [D_6_]DMSO). This spectrum was acquired at 700 MHz with a cold probe. Experimental signals marked by an asterisk are ^13^C satellites.

Figure 2 illustrates the types of unexpected line-shapes observed when two different spin species with similar chemical shifts are present and at least one of them is significantly influenced by radiation damping. In Figure 2 a, the two species have identical intensities since they correspond to the methyl doublet of isopropyl acetate. For two overlapping, broadened Lorentzian lines one would usually expect symmetrical broadening of each line, eventually leading to their coalescence and to the disappearance of the doublet structure. Instead we observe that each line is strongly asymmetric and a steep decrease in intensity occurs between them. This appearance of a dip or hole in the line shape is even more obvious in Figure 2 b, where we used a mixture of acetone and acetonitrile (40:5). Due to radiation damping broadening, the acetone resonance overlaps the acetonitrile one. Instead of observing a small peak or a shoulder at the acetonitrile chemical shift, which would be difficult to identify, one can notice an easily detectable dip, the magnitude of which is not directly correlated to the ratio of concentrations.

**Figure 3:**
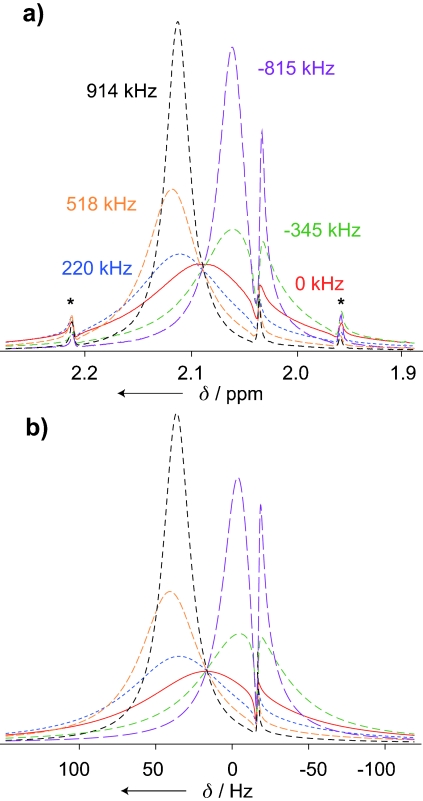
Variation of the resonance line shape of a mixture of acetone and acetonitrile (40:5) with a concentration of 80 % in volume of acetone as a function of the resonance frequency of the electronic circuit *ω*_LC_. a) Experimental spectra obtained on a 500 MHz Bruker Avance III spectrometer equipped with a TXI cryoprobe. Different colors represent different offsets Δ_LC_ relative to the condition where *ω*_LC_ matches the Larmor frequency (shown in red). For taking into account the effect of different detection sensitivities due to offset tuning, all spectra were normalized by using the DMSO signal whose concentration was sufficiently low so as to not being affected by radiation damping. Signals marked by asterisks are ^13^C satellites. b) Numerical simulations obtained by integrating Equations (10) and (11) using parameters (ℳ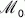, *Q*, *η*, Δ_LC_) corresponding to the experimental cases.

In order to check the consistency of the proposed interpretation, a series of 1D spectra of a mixture of acetone and acetonitrile (40:5, total acetone concentration 80 % in volume) was acquired with small flip angle excitation pulses at different setting of the resonance frequency of the rf-circuit (*ω*_LC_). The results of these experiments are shown Figure 3 a. Numerical simulations corresponding to this series of experiments are reported in Figure 3 b. They were obtained by numerically integrating the system of differential equations [Eqs. (10) and (11) expressed in the rotating frame] using parameters (ℳ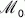, *Q*, *η*) deduced from the experimental conditions.

Several items can be seen from Figure (3):

- The comparison between the experimental and simulated data reveals near-perfect agreement. This clearly substantiates the theoretical derivation and proves that the non-linear coupling between the two magnetization densities mediated by the detection coil is the reason for observing such unexpected line shapes.
- The peak shape and position of the acetone signal are strongly dependent on the tuning conditions, varying over 30 Hz. This effect results from cavity pulling.^17^, ^18^ As a function of the amount of this contribution the resonance lines of acetone and acetonitrile are more or less overlapping. By contrast, the smaller concentration of acetonitrile reduces its magnetization and thus the amplitude of the radiation damping field [Eq. (13)]. As a consequence the position of this line is practically unaffected by cavity pulling.
- In addition to the variation of the acetone peak’s resonance frequency, its line-width is dependent on the tuning offset. It is broadest when the cavity pulling effect vanishes (red curves in Figure 3). At this condition we observed a probe-dependent offset from the point where the sensitivity of the detection circuit was previously found to be highest (the spin noise tuning optimum, SNTO).^22^, ^23^ This offset results from the fact that, while the feed-back field is governed by the flowing current inside the coil, the SNTO corresponds to a voltage optimum. The high impedance of the preamplifier then causes the difference between the two resonance situations. While these conditions allow highly sensitive detection of signals not or only moderately affected by radiation damping, an intense signal of long intrinsic transverse self-relaxation time *T*_2_ is reduced in sensitivity due to the extremely strong radiation damping. These two observations are in agreement with the fact that the induced change of Δ*ω*_LC_ affects the radiation damping rate: in a first approximation (small offsets), it broadens the line as *λ*_RD_cos*ψ*(*ω*) and shifts it by cavity pulling as *λ*_RD_sin*ψ*(*ω*), with *λ*_RD_ taken at *ω*_LC_=*γ*B_0_.
- The shape of the acetonitrile resonance line appears to be strongly dependent on that of acetone. Indeed when the latter is relatively narrow so they do not overlap, the acetonitrile resonance line is of Lorentzian shape and may be amplified, as easily observed on the ^13^C satellites. Increasing the overlap and thus the contribution from radiation damping to the broadening of the acetone line, the acetonitrile resonance changes from a peak superimposed on the acetone resonance to a dip or hole within the broad peak.
- Based on the numerical simulations under “hole conditions,” the exact resonance frequency of the minor component appears not to be equal to that of the minimum but it is slightly shifted in the direction opposite to the major peak’s maximum.

The system of non-linear equations [Eqs. (10) and (11)] is valid whatever the flip angle of the excitation pulse. In the present context, to avoid unreliable behavior of the electronic circuits (non-linearity of the excitation line due to mismatch, saturation of the preamplifier or of the ADC) we have limited the experiments to small flip-angle excitation pulses. This domain is also physically relevant because it corresponds to the one where linear-response theory is usually valid.^24^ Beyond this domain, the resonance line shapes can be calculated using Equations (10) and (11) for any flip-angle excitation pulses. Beside the severe deviations from the Lorentzian shapes due to radiation damping as already reported for a single line,^2^ this investigation reveals the appearance of the same peculiarities (amplification, asymmetric shape, dip in the line shape) due to cross-precession, which superimpose on resonance lines.

**Figure 4:**
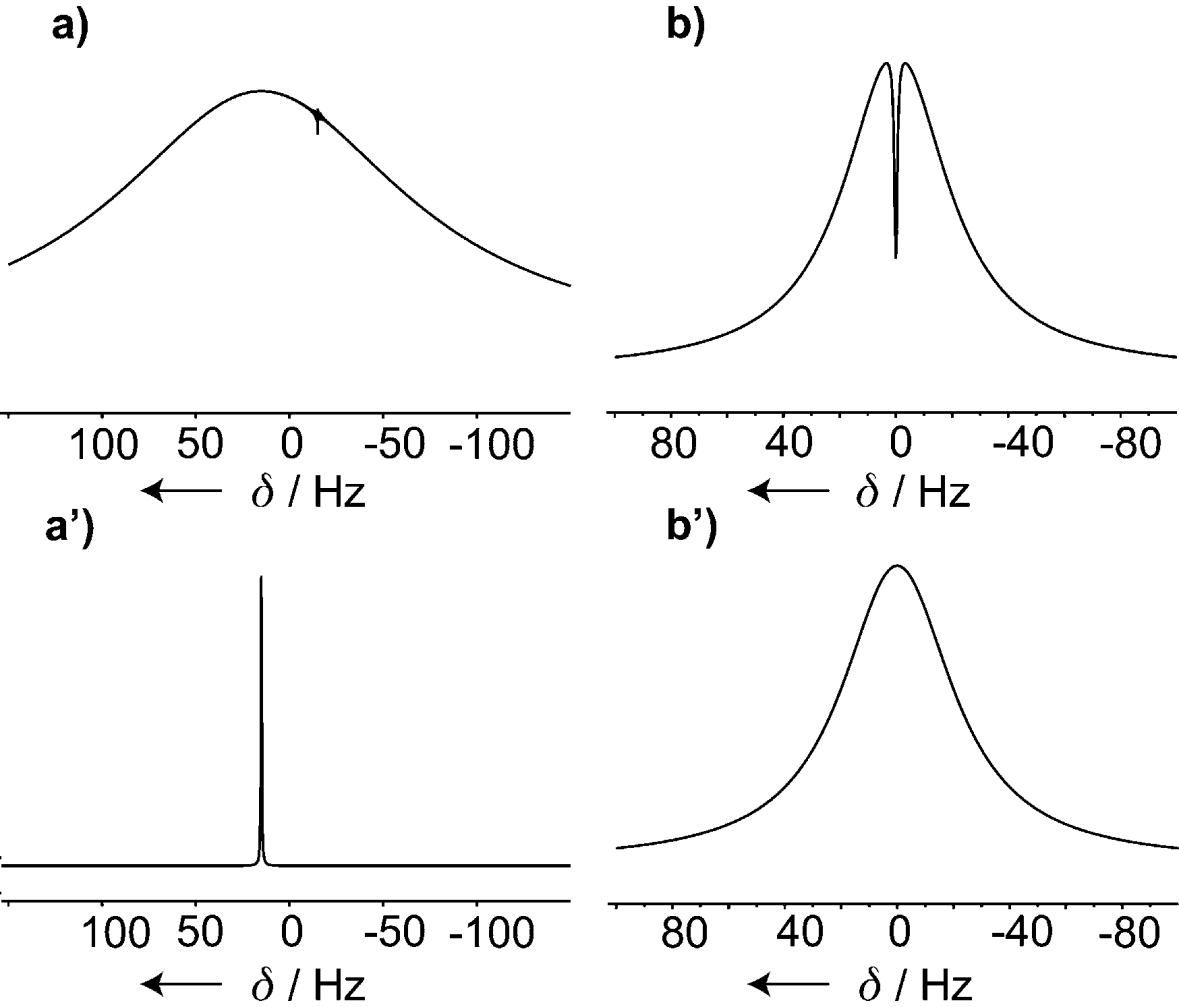
Numerical simulations of different conditions allowing one to see the influence of the existence of radiation-damping cross-precession term. a) and a′) Case of two signals with a proton ratio of 100:1. Without radiation damping (a′) the signal corresponding to the smaller signal can hardly be detected, while with radiation damping (a) this becomes possible due to the appearance of a dip in the line. b) and b′) In case of radiation damping acting on a symmetrical doublet, a hole appears between the two lines (b). In the simulations without radiation damping (b′) the two lines are broadened to give approximately the same line width as in (b) by using small values for *T*_2_. This leads to the overlap of two Lorentzian lines and the disappearance of the doublet structure.

The agreement between the experimental and simulated curves allows the evaluation of this radiation-damping cross-precession term for spectroscopic applications. Indeed, the strong distortions observed on the line shape, when resonances overlapped, in which at least one of them is affected by radiation damping, provide routes for improved detection. In Figures 4 a,a′ we illustrate this by the results obtained by numerical simulations of two species with a ratio of concentrations of 100:1. On the one hand, when one considers the system with a linear response, that is with a probe of small quality factor *Q* (Figure 4 a′), one can notice that the smallest peak is not detected. On the other hand, for a probe with a large *Q*, that is when radiation damping is present the non-linear response of the system allows simple detection of the smaller peak since a narrow distortion in the main resonance is clearly visible.

Another example of the unique features of this radiation-damping cross-precession phenomenon is provided in Figures 4 b,b′, where two resonances of identical intensity are considered. There, the transverse relaxation rates are adapted such that they exhibit the same line-widths for non-overlapping lines. In Figure 4 b′ a low-*Q* probe is assumed so that no radiation damping broadening or cross-precession is present. As one can notice, a large unstructured peak results. By contrast, in Figure 4 b, a high-*Q* probe is assumed, that is, cross-precession is now included. The line shape is strongly affected since a hole in the middle is observed. This provides a way of clearly assigning the existence of two lines, which would be rather difficult in the first case.

## 4. Conclusions

Herein, we showed that the resonance line shapes of spin systems experiencing radiation damping can exhibit severe distortions when resonances overlap. In particular, instead of the expected superposition of positive peaks when two lines overlap, one can observe a significant dip within the line shape. This previously unknown effect is explained by the fact, that through the detection coil, the precessing magnetization densities create a feedback field, which is experienced by all resonances. As a result cross-precession terms dependent on the different magnetization amplitudes have to be included in the Bloch description of the spin dynamics. These terms become non-secular when the resonance frequencies are sufficiently different. The deduced non-linear differential equations are able to represent all the experimentally observed behaviors. The discovery of this unexpected effect and its theoretical interpretation obviously raises the question of its practical application. First, the incidence of radiation-damping-broadened lines is expected to increase in the near future due to the availability of cold probes, which have very large quality factors *Q* and due to the increase of the magnetization achievable by high magnetic fields and/or by hyperpolarized species. Second, as illustrated by the simulations shown in Figure 4, the non-linear response induced by this cross-precession effect allows the improvement of detection capabilities through spectral features (existence of a doublet, tiny signals, etc.) which would have been nearly undetectable in the linear regime.

## Experimental Section

All experiments reported herein were performed at room temperature using Bruker Avance spectrometers running at 500, 600 or 700 MHz equipped with cryoprobes. Similar spectra were also obtained with classical room-temperature probes. Samples were composed of mixtures of different solvents of analytical grade. Deuterated DMSO was also added for field frequency locking and to control total spin densities. 1D NMR spectra were acquired using small flip-angle excitation pulses (<15°).

The spectral simulations were obtained, using Scilab software, by Fourier transforming the simulated time-domain signal amplitudes, which were computed by numerically integrating the system of Equations (10) and (11) expressed in the rotating frame. In order to have simulated data as closely comparable as possible to the experimental ones, the different parameters were adjusted to the respective experiments. In particular the proton concentrations needed for computing the total magnetization were those of the solution, the quality factor *Q* was set equal to 550 for the 500 MHz spectrometer in agreement with cavity-pulling experiments,^18^ and direct *Q* measurements performed with a spectrum analyzer. From the experimental line-width a value of *λ*_RD_ of 62 Hz on the 500 MHz and from the *Q* value and the magnetization, a filling factor *η* of 0.03 was deduced. The latter value is in agreement with previous reports of filling factors for saddle coil probes.^25^, ^26^

## Footnotes

1 Note that contrary to ref. 3 we explicitly consider the effect of different resonance frequencies in this derivation, which is needed due to the reduced amplitude of .
